# Depression, anxiety and suicidal ideation among 1^st^ and 2^nd^ generation migrants - results from the Gutenberg health study

**DOI:** 10.1186/s12888-016-0995-2

**Published:** 2016-08-12

**Authors:** Manfred E. Beutel, Claus Jünger, Eva M. Klein, Philipp Wild, Karl J. Lackner, Maria Blettner, Mita Banerjee, Matthias Michal, Jörg Wiltink, Elmar Brähler

**Affiliations:** 1 Department of Psychosomatic Medicine and Psychotherapy, University Medical Center Mainz, Untere Zahlbacher Str. 8, D-55131 Mainz, Germany; 2 Medical Clinic for Cardiology, Angiology and Intensive Care Medicine, University Medical Center Mainz, Mainz, Germany; 3 Preventive Cardiology and Preventive Medicine, Department of Medicine 2, University Medical Center Mainz, Mainz, Germany; 4 Center for Thrombosis and Hemostasis, University Medical Center Mainz, Mainz, Germany; 5 German Center for Cardiovascular Research (DZHK), partner site Rhine Main, University Medical Center Mainz, Mainz, Germany; 6 Institute for Clinical Chemistry and Laboratory Medicine, University Medical Center Mainz, Mainz, Germany; 7 Institute for Medical Biostatistics, Epidemiology and Informatics (IMBEI), University Medical Center Mainz, Mainz, Germany; 8 Department of English and Linguistics, American Studies, Center for Comparative Native and Indigenous Studies, Mainz, Germany

**Keywords:** Migration, Mental health, Suicidal ideation, Turkish, Polish

## Abstract

**Background:**

Even though migrants constitute a large proportion of the German population, there is a lack of representative studies on their mental health. Hence, the present study explored mental health characteristics and suicidal ideation comparing 1^st^ and 2^nd^ generation migrants to non-migrants and subgroups within 1^st^ generation migrants.

**Methods:**

We investigated cross-sectional data of 14,943 participants of the Gutenberg Health Study (GHS), a population-based, prospective, single-center cohort study in Mid-Germany (age 35 to 74 years). Migration status was assessed according to the German microcensus criteria. Depression and anxiety were measured by the PHQ (PHQ-8, GAD-2, Panic module), social anxiety by the Mini SPIN and Distressed Personality (Type D) by the DS-14. Suicidal ideation was assessed by the single item of the PHQ-9.

**Results:**

A total of *n* = 3,525 participants had a migration background; the proportion of 1^st^ generation (immigrated after 1949) migrants was 10.6 % (2^nd^ generation 13 %). Among the 1^st^ generation migrants those with Polish (*N* = 295) and Turkish (*N* = 141) origins were the largest groups from single countries. Controlling for sex, age and socioeconomic status, 1^st^ generation migrants reported significantly more depression (OR 1.24; CI 1.01-1.52), generalized anxiety (OR 1.38; CI 1.13-1.68), panic attacks in the past 4 weeks (OR 1.43; CI 1.16-1.77); Type D (OR 1.28; CI 1.13-1.45) and suicidal ideation (1.44; CI 1.19-1.74) compared to non-migrants. The mental health of 2^nd^ generation migrants did not differ from native Germans; they had the highest socioeconomic status of the three groups. Compared to native Germans, Turkish migrants of both sexes reported more depression and panic, particularly a strongly increased suicidal ideation (OR 3.02; CI 1.80-5.04) after taking sex, age, and socioeconomic status into account. Polish migrants only reported an increased rate of suicidal ideation and Type D. Turkish migrants exceeded Polish migrants regarding depression (OR = 2.61; 95 % CI 1.21-5.67), and panic attacks (OR=3.38; 95 % CI 1.45-7.85). In the subgroup analyses years lived in Germany was not significant.

**Conclusions:**

One of few representative community studies shows that compared to native Germans depression, anxiety and suicidal ideation were more frequently reported by 1^st^ generation migrants, particularly of Turkish origin. Overall, 2^nd^ generation migrants appear to have adjusted successfully. Limitations refer to a lack of data for persons without German language skills and missing mental health data in the Turkish sample. Further analyses need to address causes of mental strains and health care needs and provision.

## Background

Migration is currently reaching a record high on a global level [1]. The proportion of migrants in Germany has risen to about 20.3 % in 2014 according to Federal census statistics [2], and keeps growing strongly. Migration is a complex process which may be voluntary or involuntary. The migratory process is generally regarded as a stressful life event [3], consisting of a stage of decision and planning (pre-migration), followed by the physical transition (migration), often characterized by multiple losses, e.g., of neighborhoods, homes, work and social ties. Even if the destination promises safety from violence and political or ethnical prosecution, escape may pose many imponderables including threats to the lives of the migrant, separation from his or her next-to kin and unforeseen losses. Described as post-migration, migrants need to adjust to new social and cultural contexts including learning a new language. Acculturation requires reconciling the culture of origin with the new culture. This often lengthy process is fraught with multiple stressors, e.g., uncertain approval by the authorities, lack of employment and social recognition. As a fourth stage, sub-standard living and minority discrimination are discussed as possible long-term outcomes [1].

While international studies have demonstrated mental and physical strains among migrants, it has remained an issue of debate if migration is associated with stress and compromised physical and mental health in the long run. In a population- based German survey, Glaesmer et al. [4] found no evidence for increased mental disorders in migrants. However, their sample of immigrants was small. In their systematic review of 34 studies, Lindert et al. [5] found increased rates of depression (44 %), respectively anxiety (44 %) only among compelled refugees; prevalence rates of labor migrants were in the range of German population samples (20 % depression; 21 % anxiety). In the WHO/EURO Multicentre Study on Suicidal Behavior, the largest available European database, suicide attempt rates were higher in about half the immigrant groups studied, and rarely lower compared to the host populations. Turks also showed higher rates despite low suicide rates in their home country [6]. Suicide attempts in women of Turkish origin in Germany were much higher compared to native German women [7], however, German data are scarce [8].

In order to determine the risk of mental disorders in migrants, risk factors such as female sex, low income and poor neighborhoods [9], time since migration [1] and ethnic group need to be taken into account. In the state of Rhineland-Palatinate the proportion of migrants has been estimated at 20.3 %. As in Germany, Turkish were the largest, mostly Muslim group of migrants. Studies have compared Turkish to Polish migrants, the largest group from Eastern Europe with a Christian background. A small study by Mewes at al. [10] showed no differences between the mental health of migrants from Turkey, the largest group of migrants in Germany, from Eastern Europe and the former Soviet Union. Wittig et al. [11], however, reported increased anxiety, depression and somatization as well as less mental health care utilization in Vietnamese and Polish migrants compared to the German population. Morawa and Erim [9] found that 109 Turkish migrants recruited from the community reported more depression and lower quality of life than a random sample of Polish migrants, who were comparable to reference values from the German population. In a primary care sample Turkish patients reported higher depression and somatoform complaints compared to German natives (when adjusted for age and sex; [12]). Comparatively strong perceived discrimination, i.e., an unequal, disadvantaging or segregating treatment, was identified as one factor which might be responsible for low mental health among Turkish migrants in a German representative sample [13]. As Turkish migrants are experienced as more culturally different than other groups, e.g., Polish migrants, they may sustain more discrimination and hence less subjective mental [14, 15].

Overall, mental health has been understudied in German migrants. Due to difficult access for physical, cultural or language reasons, studies are usually small, often based on non-random samples (e.g., patients from a certain medical practice). Different definitions of migration were used and sometimes detailed inquiries were avoided for fear of stigmatizing clients. Measures have usually been developed and validated in different populations. Migration as a risk factor turned out to be hard to disentangle from macroeconomic conditions and resulting individual opportunities [5].

The purpose of this study was to determine (1) if 1^st^ and 2^nd^ generation migrants differ from native Germans and (2) if Turkish and Polish 1^st^ generation migrants as the two largest migrant groups from single countries in our sample differ from non-migrants regarding mental health in a large and population-based survey. As indicators for mental health we used a range of brief and validated self-report measures of depression, generalized anxiety, panic, social phobia, suicidal ideation and distressed personality (Type D).

## Methods

### Study sample

The analysis was based on 3,525 migrants out of cross-sectional data of *n* = 14,943 participants enrolled in the Gutenberg Health Study (GHS) from April 2007 to April 2012 [16] who completed the questions about migration. The GHS is a population-based, prospective, observational single-center cohort study in the Rhein-Main-Region in western Mid-Germany. The GHS and its procedure, including the present study, were approved by the local ethics committee of the Medical Chamber of Rhineland-Palatinate, Germany (reference no. 837.020.07; original vote: 22.3.2007, latest update: 20.10.2015). Participation was voluntary and written informed consent was obtained from each subject upon entry into the study. The primary aim of the study was to evaluate and improve cardiovascular risk stratification. The sample was drawn randomly from the local registry in the city of Mainz and the district of Mainz-Bingen. The sample was stratified 1:1 for gender and residence (city of Mainz vs. district of Mainz-Bingen) and in equal strata for decades of age. Inclusion criteria were age 35 to 74 years and written informed consent. Persons with insufficient knowledge of German language, or physical and mental inability to participate were excluded. Based on the interim analysis 5.8 % were excluded because of the exclusion criteria. The response rate (defined as the recruitment efficacy proportion, i.e., the number of persons with participation in or appointment for the baseline examination divided by the sum of number of persons with participation in or appointment for the baseline examination plus those with refusal and those who were not contactable) was 60.3 %. A total of 14,943 participants were analyzed.

Migration was defined according to the definition of the German microcensus. We therefore included all those who migrated to the area of the Federal Republic of Germany after 1949 (1^st^ generation migrants), all non-German citizens born in Germany and all citizens born in Germany with at least one migrated parent or a parent abroad (as 2^nd^ generation migrants). Migrants came from 102 countries. The largest groups from single countries came from Poland, followed by Turkey. As their numbers were too small for analysis, other countries were combined following the procedure of the German Health Interview and Examination Survey for Children and Adolescents ((KIGGS; [17]). Thus, migrants came from middle and southern Europe (*n* = 386; 24.6 %); Poland (*n* = 295; 18.8 %); western Europe, USA, Canada, Israel and Australia (*n* = 282; 18.0 %); Turkey (*n* = 141, 9.0 %); eastern Europe and Russia (*n* = 128; 8.1 %); Arabic-Islamic countries (*n* = 125; 8.0 %); middle and south America (*n* = 44; 2.8 %); Asia (*n* = 149; 9.5 %) and Africa (*n* = 21; 1.3 %). 72.8 % of the 1^st^ generation migrants in our sample came to Germany between 1970 and 1999; 21.2 % came between 1950 and 1969, and 6 % came between 2000 and 2012.

### Materials and assessment

The 5-hour baseline-examination in the study center comprised evaluation of prevalent classical cardiovascular risk factors and clinical variables, a computer-assisted personal interview including demographic information, laboratory examinations from a venous blood sample, blood pressure and anthropometric measurements. Obesity was defined as a body-mass index ≥30 kg/m^2^. The smoking status was determined by a structured interview about different types of smoking. Current smokers are defined as smoking >1 cigarette/day or >7 cigarettes/week. In general, all examinations were performed according to standard operating procedures by certified medical technical assistants.

### Questionnaires

Depression was measured by the Patient Health Questionnaire (PHQ-8), which quantifies the frequency of being bothered by 8 diagnostic criteria of Major Depression over the past 2 weeks. Responses are summed to create a score between 0 and 24 points. As in the other PHQ scales and items below, subjects answer with 0 = “not at all”, 1 = “several days”, 2 = “over half the days”, and 3 = “nearly every day”. A PHQ-8 sum score of ≥ 10 was used for the definition of caseness for depression yielding a sensitivity and a specificity of 88 % for major depression for the German version [18, 19]. It has been shown to have good cultural equivalence [20].

Suicidal ideation is a major risk factor for suicide attempts. According to the recent studies of Goldney et al. [21] and Ladwig et al. [22] suicidal ideation was assessed by the item “In the last 2 weeks, have you had thoughts that you would be better off dead or of hurting yourself in some way?” of the depression module of the Patient Health Questionnaire (PHQ-9; [18, 23]). Subjects were identified as cases with suicidal ideation if they indicated that they were bothered by suicidal ideation at least for several days over the past two weeks.

Generalized anxiety was assessed with the two screening items of the short form of the GAD-7 (Generalized Anxiety Disorder [GAD]-7 Scale) [24]: “Feeling nervous, anxious or on edge” and “Not being able to stop or control worrying”. A sum score of 3 and more (range 0–6) out of these two items indicates generalized anxiety with good sensitivity (86 %) and specificity (83 %) for the German version. Both the GAD-7 and its two core items (GAD-2) have been shown to perform well as a screening tool for all anxiety disorders [24].

Panic disorder was screened with the brief PHQ panic module. Caseness was defined if at least two of the first four PHQ panic questions are answered with “yes”. Based on the PHQ Löwe et al. [25] also tested different algorithms for sensitivity and specificity to detect panic disorder in a large clinical German sample (*N* = 998). For a maximum of sensitivity (91 %) and specificity (88 %) they recommend the use of a modified panic algorithm (at least two of the first questions answered with “yes”).

The German version of the Mini-Social Phobia Inventory (Mini-Spin; [26]) was used to detect social anxiety. Its three items are supposed to separate between individuals with generalized social anxiety disorder and controls: “Fear of embarrassment causes to avoid doing things or speaking to people”, “I avoid activities in which I am the center of attention”, and “Being embarrassed or looking stupid are among my worst fears”. The 5-point-Likert rating scale ranges from 0 = “not at all” to 4 = “extremely”. Utilizing a cut-off score of 6 (range 0–12), the German version of the Mini-Spin is supposed to separate between individuals with generalized social anxiety disorder and controls with good sensitivity (89 %) and specificity (90 %) [26, 27].

Distressed Personality (Type D) was assessed with the German version of the DS-14 [28]. It comprises two subscales, 7 items for negative affectivity (NA) and 7 items for social inhibition (SI) to be answered on a 5-point- Likert scale from 0 (false) to 4 (true). The internal consistency of the DS-14 subscales is high (NA Cronbach’s α = 0.87, SI Cronbach’s α = 0.86). The Type D personality is defined as a pattern consisting of significant negative affectivity (NA ≥ 10) in conjunction with significant social inhibition (SI ≥ 10).

The socioeconomic status (SES) was defined according to Lampert’s and Kroll’s scores of SES with a range from 3 to 27 (3 indicates the lowest SES and 27 the highest SES) [29].

### Statistical analysis

Variables were reported as absolute numbers, percentage or mean with standard deviation or medians with 25^th^ and 75^th^ percentiles as appropriate. In order to adjust for demographic differences, we entered sex (women), age (years), socioeconomic status (range from 3 to 27) and years lived in Germany in addition to 2^nd^, respectively 1^st^ generation migrants vs. native Germans in logistic multivariable regression analyses, as predictors of mental health characteristics. The odds ratios were adjusted for possible confounding factors with logistic-regression analysis and tested statistically by Wald chi-square statistics. All reported *p*-values corresponded to 2-tailed tests; chi^2^ and Kruskal-Wallis test for 3 groups were applied as appropriate. Comparing subgroups of Turkish and Polish migrants vs. non-migrants, we also added year spent in Germany into the multivariable analysis. As this is an explorative study no adjustments for multiple testing have been done. P-values were given for descriptive reasons only. Due to the large number of tests, p-values should be interpreted with caution and in connection with effect estimates. Statistical analyses were performed using SAS for Windows 9.4 TS Level 1 M1 (SAS Institute Inc.) Cary, NC, USA.

## Results

### Mental disorders in 1^st^ vs. 2^nd^ generation migrants vs. native Germans

Table 1 compares the demographic characteristics between 1^st^, 2^nd^ generation migrants and native Germans.

**Table 1:** Demographic characteristics, mental health and health behavior: migrants 1^st^ and 2^nd^ generation vs. native population (reference)

	1^st^ generation migrants			2^nd^ generation migrants			Native population	
	*N* = 1582 (10.6 %)			*N* = 1943 (13.0 %)			*N* = 11418 (76.4 %)	
	*M* ± *SD*; %	*n*	*p*-value^a^	*M* ± *SD*; %	*n*	*p*-value^a^	*M* ± *SD*; %	*n*
Age (years)	52.6 ± 10.6	1582	<.0001	54.7 ± 11.1	1943	.003	55.5 ± 11.1	11418
Women	50.9	1582	.217	49.4	1943	.924	49.3	11418
Partnership	84.2	1582	.002	79.6	1942	.137	81.0	11418
Socioeconomic status	12.0^b^	1553	<.0001	14.0^c^	1934	<.0001	12.0^d^	11370
Employment	64.5	1565	<.0002	63.3	1935	.002	59.6	11383
Pension	21.1	1562	<.0001	30.2	1926	.035	32.7	11362
Net income <750 €	3.3	1392	<.0001	2.1	1793	.165	1.6	10569
Net income 750–1499 €	15.3	1392	<.0001	8.2	1793	.039	9.7	10569
Net income 1500–2999 €	44.7	1392	<.0001	33.8	1793	.000	38.3	10569
Net income 3000–4999 €	27.7	1392	<.0001	37.3	1793	.021	34.4	10569
Net income ≥5000 €	9.1	1392	<.0001	18.7	1793	.003	15.9	10569
Smoker	22.4	1574	<.0001	21.5	1941	.003	18.6	11405
BMI (kg/m^2^)	27.5 ± 4.7	1582	.104	27.2 ± 5.1	1943	.035	27.4 ± 5.0	11414
Alcohol gram/day	6.3 ± 12.5	1574	<.0001	11.8 ± 17.2	1941	.893	11.7 ± 17.2	11399
Current depression (PHQ-8 ≥ 10)	10.1	1440	.000	7.3	1925	.412	7.4	11310
Generalized anxiety (GAD) ≥ 3	9.5	1425	<.0001	6.4	1918	.781	6.2	11255
Panic attack (past 4 weeks)	8.3	1393	<.0001	4.8	1869	.263	5.4	11000
Social Phobia (Mini-Spin ≥ 6)	4.6	1418	.135	3.4	1918	.478	3.8	11264
Suicidal thoughts	10.9	1415	<.0001	7.0	1913	.582	7.4	11251
Type D	30.0	1415	<.0001	21.4	1923	.057	23.4	11284
Antidepressant	6.9	1568	.014	6.2	1923	.147	5.4	11298
Anxiolytic	0.8	1568	.462	0.9	1923	.763	1.0	11298

^a^chi^2^ or Kruskal Wallis test; native Germans serve as reference

^b^range: 8.0–15.0

^c^range: 10.0–18.0

^d^range: 9.0–17.0

Chi^2^ test, resp. Mann Whitney Wilcoxon Test; native Germans served as reference group

As Table 1 shows, 10.6 % of the sample were 1^st^ and 13 % were 2^nd^ generation migrants. 1^st^ generation migrants were the youngest and non-migrants the oldest group; sex was comparable. 1^st^ generation migrants had the highest rate of partnerships. 2^nd^ generation migrants had the highest socioeconomic status. Corresponding to their respective mean ages, 1^st^ generation migrants were most frequently employed, and the native population had the highest rate of pensioners. 1^st^ generation migrants had the lowest and 2^nd^ generation migrants the highest income. Concerning health behavior, 1^st^ generation migrants had the highest and non-migrants the lowest rate of smoking; the opposite was the case for alcohol intake.

Regarding current mental health, 1^st^ generation migrants had the highest rates of depression, generalized anxiety, panic attacks, depersonalization, social phobia, suicidal ideation and intake of antidepressants (but not anxiolytics), and they had the highest mean scores in the respective scales, while the scores of 2^nd^ generation migrants and non-migrants were almost identical. As the three groups differed regarding age and SES, we performed logistic multivariable regression analyses entering 2^nd^, respectively 1^st^generation migrants vs. native Germans, sex (women), age and socioeconomic status predicting mental health characteristics.

As Table 2 shows, the mental health of 2^nd^ generation migrants was comparable to native Germans. The mental health of 1^st^ generation migrants, however, was consistently worse than among native Germans: This applied to generalized anxiety, panic, type D, and suicidal ideation. There was also a trend to higher depression, but no differences regarding social phobia. Additionally, female sex, younger age and lower socioeconomic status significantly predicted depression, generalized anxiety, panic, and social phobia. Women also reported more suicidal ideation. Mental symptoms declined with age except for suicidality. A lower SES was also associated with suicidal ideation.

**Table 2:** Mental disorders in 1^st^ and 2^nd^ generation migrants vs. native Germans: logistic multivariable regression

Variables	OR	95 % CI	*p*-value	OR	95 % CI	*p*-value
	Depression^1)^			Anxiety^2)^		
2^nd^ generation vs. native Germans	0.93	(0.76-1.14)	0.49	1.04	(0.85-1.27)	0.72
1^st^ generation vs. native Germans	**1.24**	**(1.01-1.52)**	**0.039**	**1.38**	**(1.13-1.68)**	**<0.01**
Sex (Women)	**1.49**	**(1.31-1.71)**	**<0.0001**	**1.63**	**(1.42-1.87)**	**<0.0001**
Age (years)	**0.97**	**(0.97-0.98)**	**<0.0001**	**0.97**	**(0.97-0.98)**	**<0.0001**
Socioeconomic status	**0.93**	**(0.91-0.95)**	**<0.0001**	**0.96**	**(0.95-0.98)**	**<0.0001**
	Social phobia^3)^			Type D^4)^		
2^nd^ generation vs. native Germans	0.90	(0.69-1.18)	0.46	0.92	(0.82-1.04)	0.18
1^st^ generation vs. native Germans	1.03	(0.78-1.35)	0.84	**1.28**	**(1.13-1.45)**	**<0.0001**
Women	**1.52**	**(1.27-1.81)**	**<0.0001**	1.06	(0.98-1.14)	0.16
Age	**0.96**	**(0.96-0.97)**	**<0.0001**	**0.98**	**(0.98-0.99)**	**<0.0001**
Socioeconomic status	**0.94**	**(0.92-0.96)**	**<0.0001**	**0.95**	**(0.94-0.95)**	**<0.0001**
	Panic ^5)^			Suicidal ideation^6)^		
2^nd^ generation vs. native Germans	0.90	(0.72-1.14)	0.38	0.99	(0.82-1.20)	0.94
1^st^ generation vs. native Germans	**1.43**	**(1.16-1.77)**	**<0.001**	**1.44**	**(1.19-1.74)**	**<0.001**
Women	**1.66**	**(1.43-1.93)**	**<0.0001**	**1.29**	**(1.14-1.46)**	**<0.0001**
Age	**0.98**	**(0.98-0.99)**	**<0.0001**	1.00	(1.00-1.01)	0.52
Socioeconomic status	**0.96**	**(0.94-0.97)**	**<0.0001**	**0.93**	**(0.92-0.95)**	**<0.0001**

Multivariable regression analysis comparing 2nd, respectively 1st generation migrants with native Germans; additionally, sex, age and socioeconomic status were entered into the equation; *OR* odds ratio, *95 % CI* 95 % confidence interval

^1)^ c-statistic = 0.628; ^2)^ c = 0.623; c-statistic =0.615 ^4)^ c = 0.585; ^5)^ c = 0.611; ^6)^ c = 0.603; significant OR’s in bold type

### Mental disorders in Turkish and Polish 1^st^ generation migrants vs. native Germans

Table 3 compares subgroups of Turkish and Polish 1^st^ generation migrants to native Germans. Turkish migrants were youngest, were less frequently female (37.6 %), had high employment, low pension and reported low income and SES. They had lived for a longer time in Germany compared to Polish migrants (31.4 years vs. 28.2 years; Kruskal-Wallis test, *p* < .00001). They smoked almost twice as frequently as the other two groups, but consumed the least alcohol. Polish 1^st^ generation migrants more closely resembled the native Germans (age, sex distribution, SES, health behavior). However, they worked more often but reported a lower income.

**Table 3:** Demographic and health characteristics among 1^st^ generation migrants: Turkish and Polish 1^st^ generation migrants vs. native Germans

	1^st^ generation			1^st^ generation			Native population	
	Turkish Migrants			Polish Migrants				
	*N* = 141^a^			*N* = 295			*N* = 11418	
	*M* ± *SD*; %	*n*	*p*-value^b^	*M* ± *SD*; %	*n*	*p*-value^a^	*M* ± *SD*; %	*n*
Age (years)	48.3 ± 9.2	141	<.0001	53.0 ± 11.2	295	.000	55.1 ± 1.1	11418
Women	37.6	141	.006	52.2	295	.323	49.3	11418
Partnership	87.9	141	.036	84.1	292	.183	81.0	11418
Socioeconomic status	9.3 ± 4.8	135	<.0001	12.0 ± 4.2	292	<.0001	12.0 ± 4.6	11383
Employment	69.1	139	.024	68.5	292	.002	59.6	11362
Pension	14.5	138	<.0001	22.9	292	.000	32.7	10569
Net income <750 €	5.3	113	.002	1.5	267	.887	1.6	10569
Net income 750–1499 €	25.7	113	<.0001	8.6	267	.538	9.7	10569
Net income 1500–2999 €	43.4	113	.273	50.2	267	<0.0001	38.3	10569
Net income 3000–4999 €	20.4	113	.002	30.7	267	.204	34.4	10569
Net income ≥5000 €	5.3	113	.002	9.0	267	.002	15.9	11418
Years lived in Germany	31.4 ± 8.4		n.a.	28.2 ± 12.9		n.a.	n.a.	
Smoker	36.2	141	<.0001	20.1	293	.504	18.6	11418
BMI (kg/m^2^)	28.8 ± 4.5	141	<.0001	27.4 ± 4.8	295	.954	27.4 ± 5.0	11418
Alcohol gram/day	1.7 ± 5.7	141	<.0001	7.8 ± 12.9	293	<.0001	11.7 ± 17.2	10826
Current depression (PHQ-9 ≥ 10)	21.6	98	<.0001	9.6	272	.0754	6.8	10826
Generalized anxiety (GAD) ≥ 3	14.6	96	.001	8.6	280	.114	6.2	11255
Panic attack (past 4 weeks)	16.7	96	<.0001	7.4	271	.167	5.4	11000
Social Phobia (Mini-Spin ≥ 6)	10.3	97	.001	5.0	279	.283	3.8	11264
Suicidal thoughts	22.1	95	<.0001	13.2	280	.000	7.4	11251
Type D	39.6	96	.001	37.3	279	< .0001	23.4	11284
Antidepressant	5.7	140	.859	5.5	291	.925	5.4	11298
Anxiolytic	0.0	140	.245	1.4	291	.471	1.0	11298

^a^detailed numbers in the table (missing data)

^b^chi^2^ or t Kruskal Wallis test; native Germans serve as reference

With 22.4 % Turkish migrants had more than twice the rate of depression compared to Polish migrants (9.6 %), who resembled rather the native Germans regarding depression (6.8 %) and other mental health problems. Similarly, they had by far the highest rates of generalized anxiety and panic attacks. While 22.1 % of the Turkish migrants reported suicidal thoughts, these were 13.2 % among Polish and 7.4 % among the Germans. Yet, the intake of antidepressants corresponded to the Polish and native Germans. There was a considerable rate of missing data among participants with Turkish origin regarding income, and even more regarding distress and suicidality up to 32.6 %. Missing data were particularly frequent in older vs. younger Turkish participants (*p* < .001), but not related to gender. It was further increased in those on pension and Turkish migrants with a low social status. 1^st^ generation Turkish women had by far the highest rate of depression (35.1 %; Turkish men: 14.8 %); suicidal ideation (28.6 %; men: 18.3 %), panic attacks (25 %; men 11.7 %). No difference was found regarding Type D with 38.9 % (men: 40 %); generalized anxiety (16.7 %; men: 13.3 %) and Social phobia (8.1 % vs. men 11.7 %).

In a multivariable approach (Table 4), Turkish migrants consistently reported more depression, panic and suicidal ideation compared to native Germans. Compared to native Germans, Polish migrants had increased type D scores and more suicidal ideation, but otherwise no indicators of heightened distress. Additionally, women had a higher risk for depression, GAD, panic and suicidality, and there was a negative association of lower social status to type D and suicidality.

**Table 4:** Mental disorders in Turkish, respectively Polish 1^st^ generation migrants vs. native Germans: logistic multivariable regression

Variables	OR	95 % CI	*p*-value	OR	95 % CI	*p*-value
	Depression			Anxiety		
Turkish vs. native German	**2.40**	**(1.40–4.13)**	**0.0015**	1.77	(0.97**–**3.23)	0.0643
Polish vs. native German	1.22	(0.80**–**1.86)	0.36	1.20	(0.78**–**1.87)	0.40
Sex (Women)	**1.54**	**(1.32–1.79)**	**0.0000**	**1.64**	**(1.41–1.92)**	**0.0000**
Age (years)	**0.97**	**(0.97–0.98)**	**0.0000**	**0.97**	**(0.97–0.98)**	**0.0000**
Socioeconomic status	**0.97**	**(0.97–0.98)**	**0.0000**	**0.96**	**(0.94–0.98)**	**0.0000**
	Social phobia			Type D		
Turkish vs. native German	1.68	(0.82**–**3.41)	0.15	1.52	(0.99**–**2.32)	0.0530
Polish vs. native German	1.07	(0.60**–**1.89)	0.82	**1.79**	**(1.39–2.30)**	**0.0000**
Sex (Women)	**1.51**	**(1.24–1.84)**	**0.0000**	1.05	(0.96**–**1.14)	0.29
Age (years)	**0.96**	**(0.95–0.97)**	**0.0000**	**0.98**	**(0.98–0.99)**	**0.0000**
Socioeconomic status	**0.94**	**(0.95–0.97)**	**0.0000**	**0.94**	**(0.93–0.95)**	**0.0000**
	Panic			Suicidal ideation		
Turkish vs. native German	**2.62**	**(1.48–4.64)**	**0.0010**	**3.02**	**(1.80–5.04)**	**0.0000**
Polish vs. native German	1.20	(0.74**–**1.93)	0.45	**1.80**	**(1.26–2.58)**	**0.0014**
Sex (Women)	**1.65**	**(1.40–1.95)**	**0.0000**	**1.20**	**(1.04–1.38)**	**0.0120**
Age (years)	**0.98**	**(0.97–0.99)**	**0.0000**	1.00	(1.00**–**1.01)	0.41
Socioeconomic status	**0.96**	**(0.94–0.98)**	**0.0000**	**0.93**	**(0.91–0.94)**	**0.0000**

Multivariable regression analysis comparing Turkish, respectively Polish with other 1st generation migrants; additionally, sex, age and socioeconomic status were entered into the equation

*OR* odds ratio, *95 % CI* 95 % confidence interval; significant OR’s in bold type

Controlling for sex, age, SES and years lived in Germany in additional multivariate tests, Turkish had more panic attacks than Polish first generation migrants (OR = 3.38; CI 1.45 7.85; *p* = .0047; depression (OR = 2.61;CI 1.21- 5.67; *p* = 0.0149), a trend to higher suicidality (OR = 1.92; CI0.94 -3.94; *p* = 0.0748) and social phobia (OR = 2.57; CI = 0.94-7.05; *p* = 0.0664. There were no differences regarding Type D or generalized anxiety. Interestingly, years spent in Germany played no role.

## Discussion

In the population-based sample, almost one fourth of participants had a migration background, slightly exceeding official estimates of the proportion of migrants in the state of Rhineland Palatinate with 20.3 % [30]. With 10.6 %, there were slightly less 1^st^ generation migrants who had immigrated since 1949 than 2^nd^ generation migrants (13.4 %) with at least one migrated parent or a parent abroad. Migrants came from over 100 countries. Migrants with Polish and Turkish origins were the largest groups from single countries among 1^st^ generation migrants, of whom the great majority had immigrated between 1970 and 1999. We found considerable demographic and social differences between 1^st^, 2^nd^ generation migrants and native Germans. First generation migrants were the youngest group, had the highest rate of partnerships and earned least; second generation migrants had the highest socioeconomic status and income. Findings regarding health behavior were mixed: 1^st^ generation migrants reported the highest and native Germans the lowest rate of smoking; the opposite was the case for alcohol intake.

Given the considerable social group differences, we compared mental health indicators between groups adjusting for age, sex and socioeconomic status. Consistent with previous literature [9, 11] 1^st^ generation migrants reported considerably more depression, generalized anxiety, panic attacks in the past 4 weeks, and suicidal ideation compared to native Germans. 2^nd^ generation migrants did not differ from native Germans regarding mental health. We are not aware of literature on Type D; which was also increased among 1^st^ generation migrants.

Among the first generation migrants, a Turkish origin was found as a risk factor for depression and panic compared to native Germans. The heightened distress of Turkish migrants was not reflected in their comparatively low intake of antidepressants of anxiolytics, which are usually first line treatments of anxiety and depression in Germany. Compared to Polish migrants only showed an increased type D behavior and more suicidal ideation, but no other indicators of heightened distress.

These findings corresponded to Morawa and Erim [9] and Igel et al. [13] who also found that Turkish migrants reported more depression and lower quality of life compared to Polish migrants (contradictory findings by Mewes et al. [10]). They were robust after adjusting for sex, age, SES and years in Germany. Group differences in our sample were quite large (e.g., OR 3.02; 95 % CI 1.80-5.04 for suicidal ideation of Turkish vs. native Germans; OR 1.80; 95 % CI 1.26-2.58 for Polish migrants). Our findings support the high mental distress and suicidality reported in Turkish women and extends these to Turkish men who have not been included in the previous studies [8]. It is also worth noting that Polish migrants reported more Type D behavior, which is seen as a significant risk factor for impaired physical and mental health for patients with cardiovascular disease [31].

Interestingly, 2^nd^ generation migrants did not differ from native Germans and even exceeded their mean socioecomomic status, while the mental health of 1^st^ generation migrants was compromised compared to native Germans and 2^nd^ generation migrants. This finding is highly relevant, given the fact that the average time lived in Germany was about 30 years. Contradicting the phase model of Butler et al. [1], mental health problems in 1^st^ generation migrants did not dissipate with time; thus, other factors (e.g., experiences of discrimination, language problems) may be responsible [13].

Differences between migrants of Turkish and Polish vs. other countries of origin are consistent with the distinction between “visible” and “invisible minorities” [32–34] as elaborated in a North American context [16–18]. It was shown to make a difference whether the migrant is visibly different in the sense that his phenotype marks him as being different from the majority society of the country he has migrated to. In Canada, for instance, Indian, African and Caribbean migrants are said to be part of visible minority communities. Others who are not visibly different from the majority population have the privilege of “passing”; they are marked as migrants only by differences in dress, language, accent, or behavior. It has been argued that for visible minority groups, it is much harder to assimilate since, regardless of the extent to which they have adopted the cultural traits of their host societies, they often continue to be stigmatized as “foreign.” Moreover, they may have internalized the idea that their phenotype marks them as inferior to the dominant culture. Conversely, invisible minorities may be accepted into the host society much more readily, as could be the case for migrants of Polish origin. Another related issue is the concept of a “racialization of religion” [19]: In this process, a given cultural and religious difference results in the respective group being perceived as phenotypically different; cultural difference gives rise to a perception of the individual as physically different, regardless of their dress, customs, etc. This may have significant result for the individual’s self-esteem and the sense that they are being discriminated against by the majority population. Turkish migrants are frequently visibly different from the German majority society, whereas Polish migrants may have the privilege of “passing”. Regardless of the extent to which they have adopted the cultural traits of the German societies, Turkish migrants may thus continue to be stigmatized as “foreign.” Thus, Polish immigrants, being mostly of Catholic denomination, may more easily assimilate to German majority culture; Turkish immigrants, who often have Muslim religion and cultural traditions, may be stigmatized and may find it harder to adopt German cultural traditions. Worse mental health among Turkish 1^st^ generation migrants compared to Polish and other origins is also consistent with previous findings attributing worse mental health of Turkish migrants to differences in correspondence between cultural, religious aspects and physical appearance and resulting experiences of discrimination which then contribute to decreased health [14]. While we cannot preclude language problems as a potential reason, an alternative explanation for the considerable proportion of older, low status Turkish 1^st^ generation migrants refusing to answer the mental health questions could be a culturally bound reluctance to admit mental health problems. Similar reasons could account for the comparatively low rate of antidepressant treatment among Turkish migrants. These findings underscore the need for providing suitable and culturally sensitive psychosocial services to migrants [35].

### Limitations

The main limitation of our study pertains to the cross-sectional data acquisition and the fact that only German speaking migrants were able to participate. As the proportion of migrants with insufficient language skills has been estimated at about 18 %, we cannot preclude bias in the recruitment [3]. The strengths are a) the well characterized population of participants between 35 and 74 years living in the Rhein-Main Region in Germany and b) the relatively large sample size. Unlike previous studies [9], we adjusted statistically for demographic differences between subsamples. While we could identify Turkish 1^st^ generation migrants as a risk group for distress and suicidal ideation, figures have to be interpreted with caution based on a high rate of missing data in this subgroup. This was frequently the case in older participants, again, we cannot preclude potential selection based on written language skills. Unfortunately, we did not measure discrimination. Cultural equivalence has only been established for one of our main outcome measures, the PHQ-9, so far [20]. Further analyses will further explore potential stresses contributing to the mental health problems identified.

## Conclusion

The findings of the present study indicate that 1^st^ generation migrants, particularly of Turkish origin, reported more distress than native Germans, whereas the mental health of 2^nd^ generation migrants did not differ from native Germans. Identifying subpopulations among migrants with higher levels of distress and associated risk factors is essential for providing suitable and culturally sensitive psychosocial services to migrants.
